# Access to maternal health services: geographical inequalities, United Republic of Tanzania

**DOI:** 10.2471/BLT.17.194126

**Published:** 2017-10-31

**Authors:** Claudia Hanson, Sabine Gabrysch, Godfrey Mbaruku, Jonathan Cox, Elibariki Mkumbo, Fatuma Manzi, Joanna Schellenberg, Carine Ronsmans

**Affiliations:** a Department of Disease Control, London School of Hygiene & Tropical Medicine, Keppel Street, London, WC1E 7HT England.; b Institute of Public Health, Ruprecht-Karls-University, Heidelberg, Germany.; c Ifakara Health Institute, Dar es Salaam, United Republic of Tanzania.; d Bill and Melinda Gates Foundation, Seattle, United States of America.; e Department of Infectious Disease Epidemiology, London School of Hygiene & Tropical Medicine, London, England.

## Abstract

**Objective:**

To determine if improved geographical accessibility led to increased uptake of maternity care in the south of the United Republic of Tanzania.

**Methods:**

In a household census in 2007 and another large household survey in 2013, we investigated 22 243 and 13 820 women who had had a recent live birth, respectively. The proportions calculated from the 2013 data were weighted to account for the sampling strategy. We examined the association between the straight-line distances to the nearest primary health facility or hospital and uptake of maternity care.

**Findings:**

The percentages of live births occurring in primary facilities and hospitals rose from 12% (2571/22 243) and 29% (6477/22 243), respectively, in 2007 to weighted values of 39% and 40%, respectively, in 2013. Between the two surveys, women living far from hospitals showed a marked gain in their use of primary facilities, but the proportion giving birth in hospitals remained low (20%). Use of four or more antenatal visits appeared largely unaffected by survey year or the distance to the nearest antenatal clinic. Although the overall percentage of live births delivered by caesarean section increased from 4.1% (913/22 145) in the first survey to a weighted value of 6.5% in the second, the corresponding percentages for women living far from hospital were very low in 2007 (2.8%; 35/1254) and 2013 (3.3%).

**Conclusion:**

For women living in our study districts who sought maternity care, access to primary facilities appeared to improve between 2007 and 2013, however access to hospital care and caesarean sections remained low.

## Résumé

**Objectif:**

Déterminer si l'amélioration de l'accessibilité géographique a permis d'augmenter le recours aux soins de maternité dans le sud de la République-Unie de Tanzanie.

**Méthodes:**

À l'occasion d'un recensement des ménages réalisé en 2007 et d'une autre grande enquête menée auprès des ménages en 2013, nous nous sommes intéressés respectivement à 22 243 et 13 820 femmes dont la grossesse avait récemment abouti à une naissance vivante. Les proportions calculées à partir des données de 2013 ont été pondérées afin de tenir compte de la stratégie d'échantillonnage. Nous avons examiné l'association entre la distance directe jusqu'à l'établissement de soins primaires ou l'hôpital le plus proche et le recours aux soins de maternité.

**Résultats:**

Le pourcentage de naissances vivantes survenues dans des établissements de soins primaires et des hôpitaux est passé respectivement de 12% (2571/22 243) et 29% (6477/22 243) en 2007 à des valeurs pondérées de 39% et 40% en 2013. Entre les deux enquêtes, nous avons observé une nette augmentation du recours aux établissements de soins primaires par les femmes qui vivaient loin des hôpitaux, mais la proportion de femmes à accoucher à l'hôpital est restée faible (20%). L'année de l'enquête ou la distance jusqu'au centre de soins prénataux le plus proche n'a guère changé les chiffres concernant la venue à quatre consultations prénatales ou plus. Même si le pourcentage global de naissances vivantes survenues par césarienne est passé de 4,1% (913/22 145) lors de la première enquête à une valeur pondérée de 6,5% lors de la seconde, les pourcentages correspondants pour les femmes résidant loin des hôpitaux étaient très faibles en 2007 (2,8%; 35/1254) et en 2013 (3,3%).

**Conclusion:**

Pour les femmes qui vivaient dans les districts observés et qui ont eu besoin de soins de maternité, l'accès aux établissements de soins primaires s'est amélioré entre 2007 et 2013; cependant, l'accès aux hôpitaux et aux césariennes est resté faible.

## Resumen

**Objetivo:**

Determinar si una mejora de la accesibilidad geográfica conduce a una mayor aceptación de la atención materna en el sur de la República Unida de Tanzanía.

**Métodos:**

En un censo doméstico de 2007 y otra encuesta doméstica de 2013, se investigaron a 22.243 y 13.820 mujeres que recientemente habían dado a luz a un nacido vivo, respectivamente. Las proporciones calculadas a partir de los datos de 2013 se ponderaron para tener en cuenta la estrategia de muestreo. Se examinó la asociación entre las distancias en línea recta hasta el centro de salud primaria o el hospital más cercano y la aceptación de la atención materna.

**Resultados:**

Los porcentajes de nacidos vivos que se producen en centros de atención primaria y hospitales aumentaron del 12% (2571/22.243) y el 29% (6477/22.243), respectivamente, en 2007 a valores ponderados del 39% y 40%, respectivamente, en 2013. Entre las dos encuestas, las mujeres que vivían lejos de los hospitales mostraron un aumento notable en el uso de centros de atención primaria, pero la proporción que dio a luz en hospitales se mantuvo baja (20%). El uso de cuatro o más visitas prenatales no se vio afectado en gran medida por el año de la encuesta o la distancia a la clínica prenatal más cercana. Aunque el porcentaje general de nacidos vivos por cesárea aumentó del 4,1% (913/22.145) en la primera encuesta a un valor ponderado del 6,5% en la segunda, los porcentajes correspondientes para las mujeres que viven lejos de un hospital fueron muy bajos en 2007 (2,8%; 35/1254) y 2013 (3,3%).

**Conclusión:**

Para las mujeres que viven en nuestros distritos de estudio que buscaron atención materna, el acceso a los centros de atención primaria pareció mejorar entre 2007 y 2013. Sin embargo, el acceso a la atención hospitalaria y a las cesáreas se mantuvo bajo.

## ملخص

**الغرض:**

تحديد إذا ما كان تيسير الحصول على الخدمات الصحية في المناطق الجغرافية المختلفة قد أثمر عن زيادة الاستفادة من خدمات رعاية الأمومة في جنوب جمهورية تنزانيا المتحدة.

**الطريقة:**

استعنا بتعداد للأسر تم إجراؤه في عام 2007 وكذلك دراسة استقصائية واسعة النطاق للأسر تم إجراؤها في عام 2013 للبحث في 22243 حالة و13820 حالة أخرى على التوالي من حالات النساء اللاتي رُزقن بمولود حي مؤخرًا. وتم ترجيح النسب المحتسبة اعتمادًا على بيانات عام 2013 لتناسب استراتيجية انتقاء العينات. ونظرنا في الارتباط بين مسافات المسارات المستقيمة المؤدية لأقرب مستشفى أو منشأة صحية رئيسية والاستفادة من خدمات رعاية الأمومة.

**النتائج:**

ارتفعت النسب المئوية للمواليد الأحياء في المستشفيات والمنشآت الصحية الرئيسية من 12% (2571/22243) و29% (6477/22243)، على التوالي، في عام 2007 لتصل إلى القيم المرجحة البالغة 39% و40%، على التوالي، في عام 2013. وتبين أن النساء اللاتي تعشن في مناطق بعيدة عن المستشفيات قد استفدن، في الفترة بين الدراستين الاستقصائيتين، استفادةً ملحوظة فيما يتعلق بحصولهن على خدمات المنشآت الصحية الرئيسية، بينما ظلت نسبة الولادة داخل المستشفيات منخفضة (20%). ولم يؤثر العام الذي تم فيه إجراء الدراسة الاستقصائية، أو المسافة التي تقطعها النساء للوصول إلى أقرب عيادة تقدم خدمات ما قبل الولادة، تأثيرًا كبيرًا على الاستفادة من الزيارات الصحية فيما قبل الولادة والبالغ عددها أربع زيارات أو أكثر. وعلى الرغم من أن النسبة المئوية الإجمالية للمواليد الأحياء الذين تمت ولادتهم بالجراحة القيصرية قد ارتفعت من 4.1% (913/22145) في الدراسة الاستقصائية الأولى لتبلغ القيمة المرجحة لها 6.5% في الدراسة الثانية، فإن النسب المئوية المناظرة للنساء اللاتي تعشن بعيدًا عن المستشفى كانت منخفضة للغاية في عام 2007 (2.8%؛ 35/1254) وعام 2013 (3.3%).

**الاستنتاج:**

ظهور تحسن في مستوى الاستفادة من المنشآت الصحية الرئيسية في الفترة بين عامي 2007 و2013 بالنسبة للنساء ممن يعشن في الأقاليم التي شملتها الدراسة التي أجريناها واللاتي كن بحاجةٍ إلى خدمات رعاية الأمومة، وبالرغم من ذلك فإن درجة الاستفادة من خدمات الرعاية المقدمة داخل المستشفيات والخدمات التي تقدمها أقسام الجراحة القيصرية ظلت منخفضة.

## 摘要

**目的:**

旨在确定提高地域性普及率是否能在坦桑尼亚联合共和国南部地区增加孕产妇的护理接受率。

**方法:**

在 2007 年的家庭普查和 2013 年的另一次大型家庭调查中，我们分别调查了 22 243 名和 13 820 名最近有活产的妇女。考虑到抽样策略，对 2013 年数据计算的比例进行了加权。我们研究了距离最近的基层医疗机构或医院的直线距离与孕产妇护理接受率之间的联系。

**结果:**

2007 年，基层医疗机构和医院的活产百分比分别从 2007 年的 12% (2571/22 243) 和 29% (6477/22 243) 上升至 2013 年的 39％ 和 40％。两次调查之间，远离医院的妇女在使用基层医疗机构方面显著增加，但医院的生育比例仍然很低 (20%)。按调查年份或最近的产前诊所距离评估，四次或更多的产前检查基本上不受影响。虽然通过剖腹产的活产婴儿整体百分比从第一次调查的 4.1% (913/22 145) 增加到第二次调查的加权值 6.5％，但 2007 年和 2013 年离医院较远妇女的相应百分比非常低，分别为 2.8％ (35/1254) 和 3.3％。

**结论:**

对于生活在我们的研究区域范围内且要求生育的妇女，在 2007 年至 2013 年期间，使用基层医疗机构的机会似乎有所改善，但是接受医院护理和剖腹产的人员仍然很少。

## Резюме

**Цель:**

Определить, приведет ли улучшение географической доступности к более высокой обращаемости в службы охраны материнства на юге Объединенной Республики Танзании.

**Методы:**

В ходе переписи населения в 2007 году и еще одного крупного обследования домашних хозяйств в 2013 году мы исследовали соответственно 22 243 и 13 820 женщин, которые недавно родили живых младенцев. Пропорции, рассчитанные по данным 2013 года, были взвешены для учета стратегии выборки. Мы исследовали связь между расстояниями по прямой до ближайшего медицинского учреждения по оказанию первичной медицинской помощи или больницы и обращаемостью в службы охраны материнства.

**Результаты:**

Процент случаев рождения живых младенцев в медицинских учреждениях по оказанию первичной медицинской помощи и больницах увеличился с 12% (2571/22 243) и 29% (6477/22 243) соответственно в 2007 году до взвешенных значений 39 и 40% соответственно в 2013 году. Между этими двумя обследованиями для женщин, живущих далеко от больниц, был продемонстрирован заметный рост в обращаемости в медицинские учреждения по оказанию первичной медицинской помощи, но доля родов в больницах оставалась низкой (20%). Использование четырех или более дородовых посещений в значительной степени не зависело от года обследования или расстояния до ближайшей антенатальной клиники. Несмотря на то что общий процент случаев рождения живых младенцев путем кесарева сечения увеличился с 4,1% (913/22 145) при первом обследовании до взвешенной величины 6,5% во втором, соответствующие процентные показатели для женщин, живущих далеко от больницы, были очень низкими в 2007 году (2,8%, 35/1254) и 2013 году (3,3%).

**Вывод:**

Для женщин, живущих в районах проведения наших исследований, которые обращались в службы охраны материнства, доступ к медицинским учреждениям по оказанию первичной медицинской помощи улучшился в период с 2007 по 2013 год, однако доступ к стационарной медицинской помощи и кесаревым сечениям оставался низким.

## Introduction

Given that there were an estimated 303 000 maternal deaths and 2.6 million neonatal deaths in 2015,^1^^,^^2^ we clearly need better ways to reach mothers and their babies with effective interventions.^3^ Distance to care is known to influence uptake of health services.^4^ As the technology for geospatial measurement becomes more widely available, there has been an increasing number of studies, in low- and middle income countries, in which the association between distance to care and either uptake of care or mortality has been investigated.^5^ In general, pregnant women who live far from a health facility are those least likely to have a facility delivery.^6^^–^^10^ This appears to be the situation in the United Republic of Tanzania,^11^^,^^12^ where distance to the nearest hospital has also been found to be positively correlated with direct obstetric mortality.^13^ Relatively little is known about the association between antenatal care or caesarean section and distance to the nearest facility.^14^^–^^16^

The United Republic of Tanzania has made substantial progress in reducing child mortality, but much more limited improvements in maternal health.^17^^,^^18^ Much of the success in child health has been due to strong preventive actions that have been mediated by a dense network of primary health facilities^17^^,^^19^ and supported by policies that, since the 1980s, have focused on rural public health.^20^^,^^21^ One explicit aim of the country’s recent policy on primary care is to increase access to delivery care in primary facilities – mainly by establishing one dispensary, that can provide basic antenatal, delivery, outpatient and postnatal care, for every village. Health centres, which already provide basic laboratory diagnostics and inpatient care, are progressively being upgraded so that they can also provide comprehensive emergency obstetric care.^22^ The focused antenatal care programme, which was introduced in 2002, encourages pregnant women without known risk factors to give birth in primary facilities.^23^ However, while studies have shown that uptake of intrapartum care is increasing in most parts of the United Republic of Tanzania,^18^ it is not known whether the Tanzanian women who live in remote rural areas have benefited from the policy change. We therefore examined whether – and, if so, how – over a six-year period, the relationship between uptake of maternity care and distance to a health facility had changed in five rural districts in the south of the United Republic of Tanzania. In surveys in 2007 and 2013, we quantified the effect of both the distance to the nearest primary facility – i.e. dispensary or health centre – and the distance to the nearest hospital on four key indicators of maternity care: (i) four or more visits for antenatal care; (ii) birth in a primary facility; (iii) birth in a hospital; and (iv) birth by caesarean section. By examining the interaction between distance to facility and survey year, we then examined whether changes over time in uptake of care varied by distance to a facility.

## Methods

We used information from two geo-referenced household surveys covering the same five districts in the south of the United Republic of Tanzania: (i) a census of all 243 612 households in 2007 – primarily designed to evaluate the impact of intermittent preventive treatment with antimalarials on infant survival;^24^ and (ii) a sample survey in 2013 that assessed the impact of a home-based counselling strategy on neonatal care and survival.^25^ In both surveys, the study population comprised women who had had a live birth in the 12 months before the survey and reported on uptake of pregnancy and intrapartum care.

The study area covers three districts of the Lindi region and two districts of the Mtwara region.^26^ Most of the residents of these districts are poor and live in mud-walled houses in rural villages. Between 2009 and 2013, two dispensaries in the study area were upgraded to become health centres and 14 new dispensaries were inaugurated. By 2013, the study population was served by 156 dispensaries, 15 health centres and six hospitals within the study area and by another two hospitals just outside the district boundaries. All except four of the 179 health facilities serving the study area in 2013 – i.e. two mission hospitals, one mission dispensary and one private health centre – were public facilities that provided maternal health services free of charge.^27^

In both 2007 and 2013, all eight hospitals serving the study area provided caesarean sections on a daily 24-hour basis, three of the hospitals had maternity waiting homes and all of the hospitals and seven of the health centres were equipped with ambulances. Ambulance use – e.g. for hospital referral – was, however, severely constrained by shortages of fuel, human resources and funds for repair. Although all except one of the 179 facilities offered delivery care, basic emergency obstetric care was not consistently available in the study area.^27^^–^^29^

### Data collection

The survey methods are described in detail elsewhere.^24^^,^^25^ In brief, we used a modular questionnaire, administered in Swahili, to assess coverage of essential interventions during pregnancy and childbirth. Use of personal digital assistants to collect data facilitated the checking of standard ranges, consistency and completeness at the time of data entry.^30^ Household wealth was assessed by asking each household head about household assets and housing type. We mapped the study households using a global positioning system. The positions of the relevant health facilities had been recorded in previous surveys.

In 2007, we surveyed all 243 612 households in the five study districts. In 2013, however, we sampled 169 324 households, which were selected by following a two-stage sampling survey.^25^ Using the results of the national 2012 census, in which 247 350 households were recorded in the study area, we first sampled so-called subvillages. This sampling was proportional to the number of households in each subvillage – typically about 80–100. We included all households in the subvillages with fewer than 130 households, but used segmentation for subvillages with more than 131 households.

### Outcomes and explanatory variables

Our main outcomes of interest were uptake of at least four visits for antenatal care, delivery in a health facility and delivery by caesarean section. Using a combination of coordinates and the nearstat command in Stata version 13 (StataCorp. LP, College Station, United States of America), we calculated straight-line distances between each surveyed household and: (i) the nearest antenatal clinic, which could have been in a primary facility or a hospital; (ii) the nearest primary facility offering delivery care; and (iii) the nearest hospital. We did this separately for 2007 and 2013. In the 2007 survey, we attempted to impute the coordinates of households for which no such coordinates were recorded, from the coordinates for neighbouring households. Household wealth quintiles were constructed separately for 2007 and 2013, using principal component analysis.^31^

### Statistical analysis

All analyses were conducted in Stata version 13. For the 2013 data, we accounted for the different sampling structures of the 2007 and 2013 surveys by weighting subvillages by the inverse chance of being included. The percentages reported for 2013 – but not those reported for 2007 – are therefore weighted values. For both 2007 and 2013, we assessed the effect of: (i) distance to nearest antenatal clinic on uptake of at least four visits for antenatal care; (ii) distance to nearest primary facility on delivery in a primary facility; (iii) distance to nearest hospital on hospital delivery; and (iv) distance to nearest hospital on birth by caesarean section. For the analysis of the effect of distance on delivery in a primary facility, we excluded births where a hospital was the nearest facility.

We first used generalized linear models to calculate crude prevalence ratios (cPR) with 95% confidence intervals (CI). We compared the prevalence of each indicator by increasing distance to a primary health facility or hospital and then compared the prevalence of each indicator between 2007 and 2013 within each distance group.^32^ We adjusted the crude prevalence ratios for potential confounding by the mother’s age, parity, district of residence, education, ethnic group and occupation and her household’s wealth quintile. Using multilevel logistic regression without weighting, we fitted an interaction term between distance to facility and survey year and used the likelihood ratio test to calculate a corresponding *P*-value. We also used ArcGIS version 9.2 (ESRI, Redlands, USA) to map the absolute increases in facility delivery and caesarean section by administrative ward – as percentages of the live births – between 2007 and 2013.

### Ethics

Ethical clearance was obtained from the institutional review boards of Ifakara Health Institute, and the Tanzanian National Institute of Medical Research and the ethics committees of the London School of Hygiene and Tropical Medicine and the Swiss cantons of Basel-Stadt and Basel-Land.

The study population was informed about the surveys by the local government authorities and again, one day prior interview, by a sensitizer who used information sheets in the local language. Written consent to participate was obtained from household heads and the women who answered questions about pregnancy and childbirth.

## Results

**Fig. 1:**
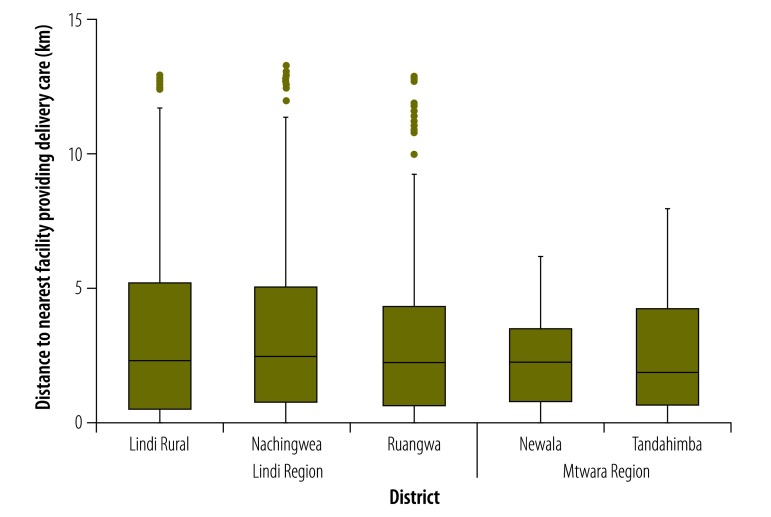
Distances to the nearest health facility providing delivery care for women in the five study districts, United Republic of Tanzania, 2013

Notes: Distances from home to the nearest facility providing delivery care were evaluated for 3114, 1564, 2437, 2907, and 3609 households in Lindi Rural, Ruangwa, Nachingwea, Newala, and Tandahimba districts, respectively. In these box-and-whisker plots, the horizontal bars, boxes, whiskers and dots indicate medians, interquartile ranges, minimum and maximum values – excluding outliers – and outliers, respectively.

**Fig. 2:**
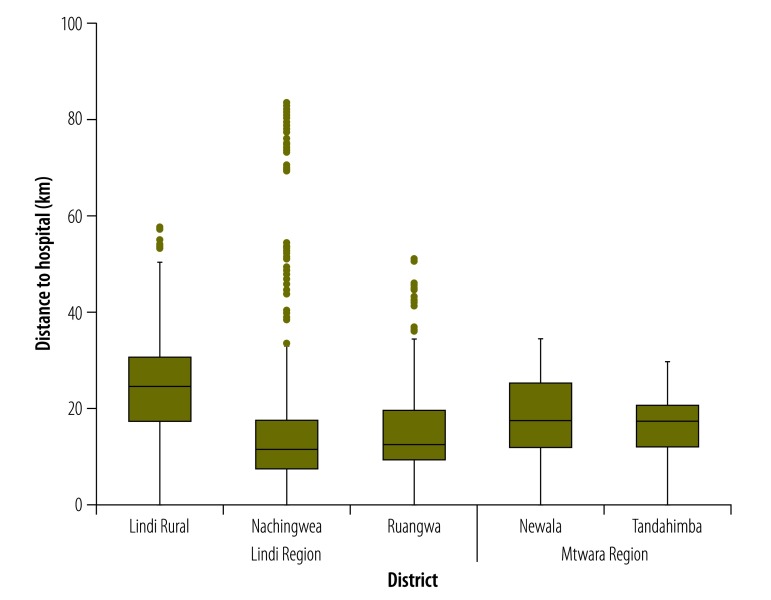
Distances to the nearest hospital providing delivery care for women in the five study districts, United Republic of Tanzania, 2013

Notes: Distances from home to the nearest facility providing delivery care were evaluated for 3114, 1564, 2437, 2907, and 3609 households in Lindi Rural, Ruangwa, Nachingwea, Newala, and Tandahimba districts, respectively In these box-and-whisker plots, the horizontal bars, boxes, whiskers and dots indicate medians, interquartile ranges, minimum and maximum values – excluding outliers – and outliers, respectively.

We conducted interviews with 321 093 consenting females who were aged 13–49 years and considered to be women of reproductive age: 193 867 in 2007 and 127 226 in 2013. Overall, 22 243 of these women had a live birth in the 12 months before the 2007 survey and 13 820 in the 12 months before the 2013 survey. Of these interviewees, 21 959 and 13 762 reported on antenatal care, 22 242 and 13 817 on place of birth and 22 145 and 13 810 on caesarean section in the 2007 and 2013 surveys, respectively. The proportions of births represented by the Mtwara region and the Makonde ethnic group were higher in the 2013 survey than in the 2007 survey (Table 1). In general, compared with those interviewed in the 2013 survey, the women interviewed in the 2007 survey were living in poorer households, less educated and of higher parity and lived further from any health facility providing delivery care in the study area (median: 2.7 km in 2007 vs 2.2 km in 2013; Fig. 1). The median distance to a hospital was 18.0 km in both surveys (Fig. 2).

**Table 1:** Characteristics of the female subjects of a two-survey study of access to maternity care, United Republic of Tanzania, 2007 and 2013

Characteristic	No. (%) of subjects**^a^**		*P*^b^
	2007 survey (*n* = 22 243)	2013 survey (*n* = 13 820)	
**Region**			< 0.001
Lindi	13 107 (59)	7131 (49)	
Mtwara	9136 (41)	6689 (51)	
**Ethnic group**			< 0.001
Makonde	11 989 (54)	8010 (60)	
Other	10 254 (46)	5804 (40)	
**Household wealth quintile^c^**			< 0.001
Most poor	3331 (15)	1804 (13)	
Very poor	3963 (18)	2556 (18)	
Poor	4631 (21)	2810 (20)	
Less poor	4710 (21)	3025 (22)	
Least poor	4722 (21)	3426 (25)	
Data missing	886 (4)	199 (2)	
**Education**			< 0.001
None	6434 (29)	2744 (20)	
Some primary	3298 (15)	1579 (11)	
Completed primary	12 367 (56)	9362 (68)	
Secondary or higher	45 (0.2)	78 (1)	
Data missing	99 (1)	57 (0.4)	
**Occupation**			< 0.001
Subsistence farmer	20 959 (94)	12 829 (93)	
Other	895 (4)	792 (6)	
Data missing	389 (2)	199 (2)	
**Parity**			< 0.001
1	5206 (23)	4252 (31)	
2–3	9835 (44)	5398 (39)	
4–6	4693 (21)	3068 (22)	
> 6	2506 (11)	1100 (8)	
**Age, years**			< 0.001
< 20	3193 (14)	2431 (18)	
20–29	10 747 (48)	6066 (44)	
30–39	6684 (30)	4189 (30)	
40–49	1619 (7)	1134 (8)	
**Distance to nearest primary facility, km^d^**			< 0.001
< 1.0	5472 (27)	4366 (34)	
1.0– < 2.5	2989 (15)	2415 (19)	
2.5– < 5.0	5663 (28)	3915 (30)	
5.0– < 7.5	2868 (14)	1681 (13)	
≥ 7.5	1056 (5)	447 (3)	
Missing data	2223 (11)	189 (2)	
**Distance to nearest hospital, km**			< 0.001
< 5.0	1838 (8)	949 (7)	
5.0– < 10.0	2420 (11)	1767 (13)	
10.0– < 15.0	3646 (16)	2684 (19)	
15.0– < 25.0	7174 (32)	5099 (39)	
25.0– < 35.0	3681 (17)	2268 (17)	
≥ 35.0	1261 (6)	864 (5)	
Missing data	2223 (10)	189 (1)	

^a^ All the subjects were women of reproductive age who reported giving birth in the 12 months before the survey. All of the percentages for 2013 were computed taking sampling weights into consideration.

^b^ For each characteristic, the significance of the between-survey difference was investigated in a *χ*^2^ test.

^c^ There are not equal numbers of subjects from each quintile because, in our study population, the mean number of women of reproductive age per household tended to increase with increasing household wealth.

^d^ Excluding the mothers whose nearest facility was a hospital.

Coverage with four or more antenatal visits increased only marginally, from 41% (9082/21 959) in 2007 to a weighted value of 45% in 2013 (cPR: 1.1; 95% CI: 1.1–1.1), and there was no association between distance to an antenatal clinic and uptake of such antenatal care in 2007 or 2013 (Table 2). Although the interaction between study year and distance to an antenatal clinic was statistically significant (*P* = 0.011), the between-survey changes seen in uptake of antenatal care in each distance category were very small.

**Table 2:** Variation in the uptake of antenatal and maternity care according to the distance to nearest primary facility or hospital at which such care was available, United Republic of Tanzania, 2007 and 2013

Type of care, distance to that care,km	Interviewees in 2007/2013**^a^**	Uptake of care in 2007/2013, %**^b^**	cPR (95% CI)			aPR(95% CI)^c^			*P***^d^**
			2007	2013		2007	2013	Change between 2007 and 2013	
**Antenatal care^e^**									0.011
< 1.0	5971/4560	44/46	Reference	Reference		Reference	Reference	1.0 (1.0–1.1)	
1.0– < 2.5	3638/2709	43/47	1.0 (0.9–1.0)	1.0 (1.0–1.1)		1.0 (0.9–1.0)	1.0 (1.0–1.1)	1.1 (1.0–1.2)	
2.5– < 5.0	6067/4133	41/45	0.9 (0.9–1.0)	1.0 (0.9–1.1)		0.9 (0.9–1.0)	1.0 (1.0–1.1)	1.1 (1.1–1.2)	
5.0– < 7.5	3001/1702	40/44	0.9 (0.9–1.0)	1.0 (0.9–1.0)		0.9 (0.9–1.0)	1.0 (0.9–1.1)	1.1 (1.0–1.2)	
≥ 7.5	1086/471	36/41	0.8 (0.8–0.9)	0.9 (0.8–1.0)		0.8 (0.7–0.9)	0.9 (0.8–1.0)	1.1 (1.0–1.3)	
Missing data	2196/187	40/38	0.9 (0.8–1.0)	0.8 (0.7–1.0)		0.9 (0.8–1.0)	0.9 (0.7–1.1)	N/A	
Total	21 959/13 762	41/45	N/A	N/A		N/A	N/A	1.1(1.1–1.1)	
**Delivery in primary facility**									< 0.001
< 1.0	5472/4364	22/50	Reference	Reference		Reference	Reference	2.3 (2.1–2.5)	
1.0– < 2.5	2989/2415	13/42	0.6 (0.5–0.7)	0.8 (0.8–0.9)		0.6 (0.5–0.7)	0.8 (0.8–0.9)	3.4 (3.0–3.9)	
2.5– < 5.0	5663/3914	8/35	0.3 (0.3–0.4)	0.7 (0.7–0.8)		0.3 (0.3–0.4)	0.7 (0.6–0.8)	4.8 (4.2–5.6)	
5.0– < 7.5	2867/1681	7/35	0.3 (0.2–0.4)	0.7 (0.6–0.8)		0.3 (0.2–0.4)	0.7 (0.6–0.8)	5.3 (4.2–6.6)	
≥ 7.5	1056/447	6/28	0.3 (0.2–0.4)	0.6 (0.4–0.7)		0.3 (0.2–0.4)	0.6 (0.5–0.7)	4.1 (2.7–6.2)	
Missing data	2223/189	12/30	0.6 (0.4–0.7)	0.6 (0.4–0.9)		0.6 (0.4–0.7)	0.6 (0.4–0.9)	N/A	
Total	20 270/13 010	13/41	N/A	N/A		N/A	N/A	3.3 (3.1–3.6)	
**Delivery in hospital**									< 0.001
< 5.0	1828/949	72/88	Reference	Reference		Reference	Reference	1.2 (1.1–1.3)	
5.0– < 10.0	2420/1767	34/57	0.5 (0.4–0.5)	0.7 (0.6–0.7)		0.6 (0.5–0.6)	0.7 (0.7–0.8)	1.6 (1.5–1.8)	
10.0– < 15.0	3646/2683	26/41	0.4 (0.3–0.4)	0.5 (0.4–0.5)		0.5 (0.4–0.5)	0.5 (0.5–0.6)	1.5 (1.4–1.7)	
15.0– < 25.0	7173/5099	22/33	0.3 (0.3–0.3)	0.4 (0.4–0.4)		0.4 (0.4–0.4)	0.4 (0.4–0.5)	1.4 (1.3–1.5)	
25.0– < 35.0	3681/2267	23/27	0.3 (0.3–0.4)	0.3 (0.3–0.3)		0.4 (0.4–0.4)	0.3 (0.3–0.4)	1.1 (1.0–1.3)	
≥ 35.0	1261/863	21/22	0.3 (0.3–0.3)	0.3 (0.2–0.3)		0.3 (0.3–0.4)	0.3 (0.2–0.3)	0.9 (0.7–1.1)	
Missing data	2223/189	30/47	0.4 (0.4–0.5)	0.5 (0.4–0.7)		0.5 (0.4–0.6)	0.6 (0.5–0.8)	N/A	
Total	22 242/13 817	29/40	N/A	N/A		N/A	N/A	1.3 (1.2–1.4)	
**Birth by caesarean section**									0.208
< 5.0	1833/949	8.0/12.6	Reference	Reference		Reference	Reference	1.5 (1.2–1.9)	
5.0– < 10.0	2415/1764	5.1/8.0	0.6 (0.5–0.8)	0.6 (0.5–0.8)		0.9 (0.7–1.1)	0.7 (0.6–0.9)	1.5 (1.2–1.9)	
10.0– < 15.0	3636/2683	3.9/6.3	0.5 (0.4–0.6)	0.5 (0.4–0.6)		0.7 (0.5–0.8)	0.6 (0.5–0.7)	1.5 (1.2–1.9)	
15.0– < 25.0	7136/5097	3.8/6.1	0.5 (0.4–0.6)	0.5 (0.4–0.6)		0.7 (0.5–0.8)	0.6 (0.5–0.7)	1.5 (1.2–1.7)	
25.0– < 35.0	3658/2264	2.9/5.3	0.4 (0.3–0.5)	0.4 (0.3–0.5)		0.5 (0.4–0.6)	0.5 (0.4–0.6)	1.8 (1.4–2.3)	
≥ 35.0	1254/864	2.8/3.3	0.4 (0.2–0.5)	0.3 (0.2–0.4)		0.5 (0.3–0.8)	0.3 (0.2–0.4)	1.0 (0.6–1.7)	
Missing data	2213/189	4.0/8.9	0.5 (0.4–0.7)	0.7 (0.4–1.2)		0.7 (0.5–0.9)	0.8 (0.5–1.4)	N/A	
Total	22 145/13 810	4.1/6.5	N/A	N/A		N/A	N/A	1.5 (1.3–1.6)	

aPR: adjusted prevalence ratio; CI: confidence interval; cPR: crude prevalence ratio; N/A: not applicable.

^a^ Women of reproductive age who reported giving birth in the previous 12 months.

^b^ All of the percentages for 2013 were computed taking sampling weights into consideration.

^c^ Adjusted for the mother’s age, parity, district of residence, education, ethnic group and occupation and her household’s wealth quintile.

^d^ For the interaction between distance to facility and survey year, as assessed in likelihood ratio tests.

^e^ At least four visits.

Table 2 summarizes the cPRs and adjusted PRs (aPR). After excluding the data for areas where a hospital is the nearest facility, the proportion of births occurring in primary facilities increased from 13% (2546/20 270) in 2007 to a weighted value of 41% in 2013 (aPR: 3.3). The proportion of births occurring in hospitals also increased, from 29% (6475/22 242) in 2007 to a weighted value of 40% in 2013 (aPR: 1.3). In both surveys, the distance to a primary facility was strongly associated with delivery in a primary facility. The between-survey increases in the proportion of births occurring in primary facilities were most pronounced among the women who lived relatively far away from a primary facility (*P* < 0.001; Table 2). For example, for those living less than 1.0 km from a primary facility, the proportion of births that occurred in such a facility increased from 22% (1219/5472) in 2007 to a weighted value of 50% in 2013 (aPR: 2.3). The corresponding values for those living at least 7.5 km from a primary facility were 6% (63/1056) and 28%, respectively (aPR: 4.1). In contrast, the between-survey increases in the proportion of births occurring in hospitals were greatest for those living at least 5.0 km, but less than 10.0 km from a hospital – 34% (824/2420) versus a weighted value of 57% (aPR: 1.6) – or at least 10.0 km but no more than 15.0 km from a hospital – 26% (960/3646) versus a weighted value of 41% (aPR: 1.5) (Table 2).

**Fig. 3:**
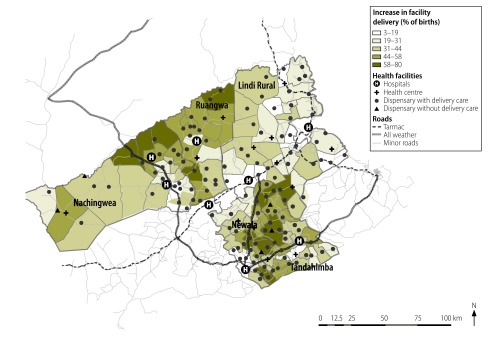
Ward map of the five study districts showing the increases in facility deliveries, United Republic of Tanzania, 2007–2013

Notes: The map, which shows the percentage increase in the proportions of births in the previous 12 months that were facility deliveries, is based on data collected in interviews with 22 243 women in 2007 and 13 833 in 2013. All of the percentages for 2013 were computed taking sampling weights into consideration.

**Fig. 4:**
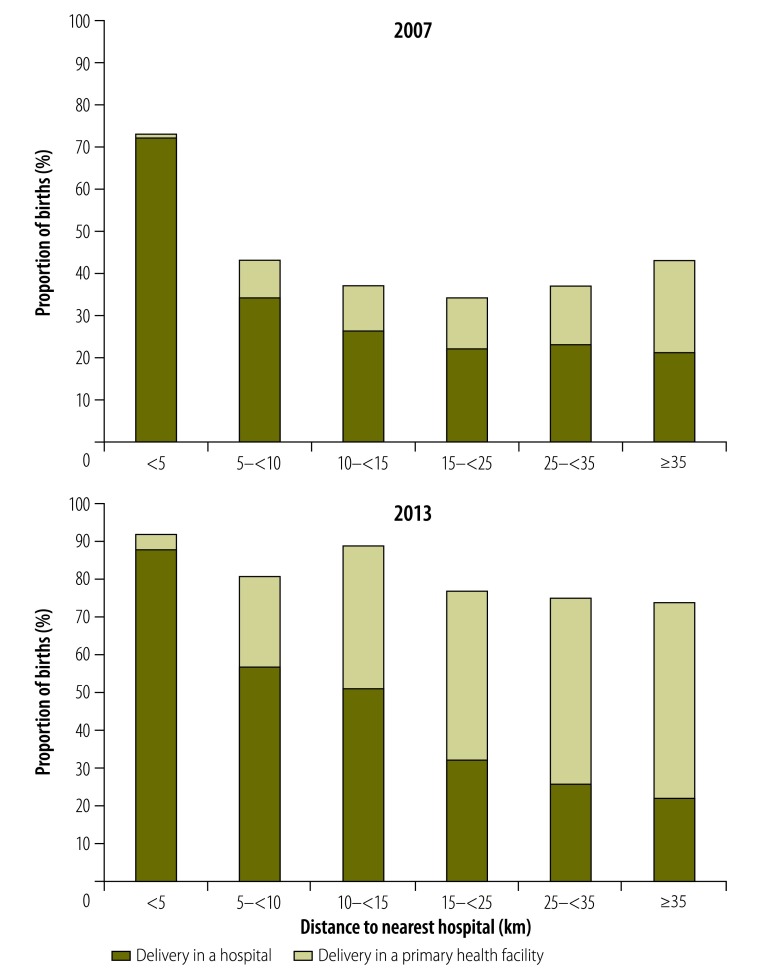
Changes in the proportions of births occurring in a health facility according to distance to nearest hospital, United Republic of Tanzania, 2007 and 2013

Note: All of the percentages for 2013 were computed taking sampling weights into consideration.

Overall, the proportion of women giving birth in any health facility, whether it was a primary facility or a hospital, increased from 41% (9021/22 242) in 2007 to a weighted value of 79% in 2013. The greatest absolute increase was seen in the rural, remote wards that were at least 10.0 km from a hospital (Fig. 3). The share of births occurring in primary facilities increased with the distance to the nearest hospital. In terms of the weighted proportions for 2013, only 4% of the women living very close to a hospital – i.e. at a distance of less than 5.0 km – gave birth in a primary facility. The corresponding proportions for the women living at least 25.0 km but less than 35.0 km and more than 35.0 km from their nearest hospital were much greater: 49% and 52%, respectively (Fig. 4).

**Fig. 5:**
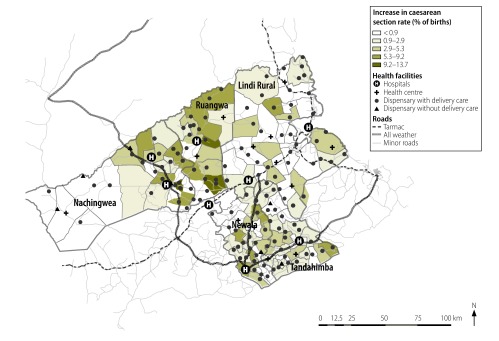
Ward map of the five study districts showing the increases in caesarean sections, United Republic of Tanzania, 2007–2013

Notes: The map, which shows the percentage increase in the proportions of births in the previous 12 months that involved caesarean sections, is based on data collected in interviews with 22 243 women in 2007 and 13 833 in 2013. All of the percentages for 2013 were computed taking sampling weights into consideration.

The proportion of births represented by caesarean sections increased from 4.1% (913/22 145) in 2007 to a weighted value of 6.5% in 2013. The level of increase in the frequency of caesarean sections appeared unaffected by the distance to the nearest hospital (*P* = 0.208; Table 2; Fig. 5) even though, in both surveys, there was a strong negative association between distance to the nearest hospital and delivery by caesarean section. For the women living more than 35.0 km from their nearest hospital, there was no between-survey increase in the proportion of births represented by caesarean sections (aPR: 1.0; Table 2).

## Discussion

The data presented here provide evidence of substantial increases, between 2007 and 2013, in the proportion of births in the study area, represented by deliveries in primary facilities and hospitals. The large increase we observed in facility births is consistent with findings from other Tanzanian studies.^18^^,^^33^^,^^34^ The increased uptake of delivery in primary facilities for women who live in the more remote areas, often far from a hospital, is particularly noteworthy. The increase probably indicates that the national policy to improve access to maternity care – by promoting delivery care in primary facilities and further increasing the number of such facilities – is being successful.^23^^,^^35^ The United Republic of Tanzania’s substantial socioeconomic development,^36^ including improvements in the road network, may have helped women to travel moderate distances while seeking maternity care. Also, two projects to support birth preparedness, at community level, may also have had a beneficial impact in the study area.^25^^,^^37^

While we may have seen an important reduction in the inequality of geographical access to primary care for childbirth, there appeared to be little between-survey improvement in access to hospital-based delivery care or caesarean sections. In our study area, the district hospitals are expected to send ambulances to dispensaries, to collect patients who need emergency hospital care. However, such emergency referrals are severely constrained by lack of funds at district level to pay for the fuel, maintenance and repairs needed to keep ambulances on the road.^37^ In addition, only three of the hospitals serving the study area had maternity waiting homes.

While the optimal caesarean section rate remains a matter of controversy,^38^^–^^41^ rates of about 3% – as seen in the more remote settings in our study area – are far too low to meet the needs of pregnant women and their babies.

The persistently low uptake of antenatal care by Tanzanian women has been noted previously.^42^ In our study, distance to a facility had no apparent effect on uptake of such care. This observation is in line with findings from Zambia,^15^ but conflicts with the results of an earlier study in the United Republic of Tanzania.^35^ However, this earlier study did not include dispensaries, which are the main providers of antenatal care in the country.^35^ As the World Health Organization has now increased the recommended number of antenatal visits to at least eight,^43^ it is, perhaps, even more important to examine the reasons for the suboptimal levels of antenatal care seen in the United Republic of Tanzania.^44^

Our study had several strengths, including its reliance on two large representative datasets from, effectively, the same study population and the use of the same questionnaire and a short recall period in both surveys. However, there may be limitations. First, the use of straight-line distance to a facility, to evaluate geographical accessibility, is sometimes regarded as inferior to calculating travel time^45^ – although this depends on the setting.^46^ The results of a Tanzanian study in which topographic maps were used to estimate travel time^47^ indicated that, at least in the United Republic of Tanzania, straight-line distances may correlate fairly well with travel times. Second, our analysis is based on a full census of the study area in 2007 but only a sample survey in 2013. Despite adjusting our estimates to take account of this difference between the surveys, we still found unanticipated and unexpected differences between the composition of the study population in 2007 and that of the study population in 2013. These differences, however, can probably be attributed to migration and other demographic changes^26^ rather than to our sampling procedure. Third, our 2007 data came from women who differed, in terms of three known drivers of the uptake of facility care –i.e. age, education and parity^48^ – from the women who provided our data in 2013. We did, however, make adjustments in our data analyses for each of these potential confounders. Lastly, we used prevalence ratios to estimate the strength of the effect of distance to the nearest facility on uptake of care. While this improves the ease of interpretation, it also increases confidence intervals.^32^

The increased uptake of facility births in our study area is encouraging. However, our analysis indicates that this increase did not translate into a substantial concurrent increase in caesarean sections. A plausible explanation is the lack of a functioning link between primary and secondary facilities, especially poor emergency referral from the primary facilities. We believe that our findings – together with existing evidence on deficits in the quality of care in primary facilities and on the high levels of neonatal mortality in our study area and other parts of the United Republic of Tanzania^25^^,^^27^^,^^28^^,^^33^ – indicate a need to revisit the policy of providing maternity care in primary facilities that are not linked to hospitals through a functioning referral system. Intrapartum care, in East Africa and elsewhere, needs to be strengthened by improvements in the quality of care and referral systems.^49^
